# ALIX Regulates the Ubiquitin-Independent Lysosomal Sorting of the P2Y_1_ Purinergic Receptor via a YPX_3_L Motif

**DOI:** 10.1371/journal.pone.0157587

**Published:** 2016-06-14

**Authors:** Michael R. Dores, Neil J. Grimsey, Francisco Mendez, JoAnn Trejo

**Affiliations:** 1 Department of Pharmacology, School of Medicine, University of California San Diego, La Jolla, CA 92093, United States of America; 2 Department of Biology, Hofstra University, Hempstead, NY 11549, United States of America; University of Geveva, SWITZERLAND

## Abstract

Endocytic sorting and lysosomal degradation are integral to the regulation of G protein-coupled receptor (GPCR) function. Upon ligand binding, classical GPCRs are activated, internalized and recycled or sorted to lysosomes for degradation, a process that requires receptor ubiquitination. However, recent studies have demonstrated that numerous GPCRs are sorted to lysosomes independent of receptor ubiquitination. Here, we describe an ubiquitin-independent lysosomal sorting pathway for the purinergic GPCR P2Y_1_. After activation, P2Y_1_ sorts to lysosomes for degradation independent of direct ubiquitination that is mediated by a YPX_3_L motif within the second intracellular loop that serves as a binding site for the adaptor protein ALIX. Depletion of ALIX or site-directed mutation of the YPX_3_L motif inhibits P2Y_1_ sorting into the lumen of multivesicular endosomes/lysosomes and degradation. These findings confirm the function of YPX_3_L motifs as lysosomal targeting sequences for GPCRs and demonstrate that ALIX mediates the ubiquitin-independent degradation of certain GPCRs.

## Introduction

G protein-coupled receptors (GPCRs) are the largest family of mammalian transmembrane signaling receptors, including over 800 members, and are important drug targets [1]. Defects in GPCR signaling have been implicated in multiple human diseases, including inflammation, vascular diseases, and cancer [2, 3]. The temporal and spatial aspects of GPCR signaling are regulated by rapid desensitization and intracellular trafficking [4, 5]. Once internalized GPCRs are either recycled back to the cell surface or sorted to lysosomes for degradation, processes important for cellular resensitization and signal termination, respectively. Consequently, disruption of GPCR lysosomal sorting can lead to aberrant signaling and disease progression [6].

The purinergic receptor P2Y_1_ is a GPCR for extracellular adenosine diphosphate (ADP), and mediates a variety of responses in distinct cell types. ADP signaling through P2Y_1_ induces mitogenesis and migration of vascular endothelial cells [7], mitogenesis of smooth muscle cells [8], platelet aggregation [9] and neuroprotective effects in astrocytes [10]. In addition, ADP stimulation of P2Y_1_ induces apoptosis in prostate cancer cells [11], suggesting that P2Y_1_ is a possible target for anti-cancer therapeutics. However, activation of P2Y_1_ also stimulates proliferation of pancreatic ductal cancer cells [12], highlighting the tissue-specificity of P2Y_1_ signaling. P2Y_1_ signaling is regulated by receptor phosphorylation and β-arrestin-mediated internalization [13]. Upon internalization from the cell surface, P2Y_1_ is efficiently recycled back to the plasma membrane, a process that is mediated by sorting nexin-1 (SNX-1) [14]. However, following prolonged ADP stimulation, P2Y_1_ is sorted from endosomes to lysosomes and degraded [14]. The mechanisms that control P2Y_1_ lysosomal sorting are poorly understood.

Given the diversity of GPCR signaling and function, multiple mechanisms exist to mediate the sorting of GPCRs to lysosomes. After activation, many GPCRs are post-translationally modified with ubiquitin, which acts as a sorting signal that is recognized by endocytic adaptor proteins containing ubiquitin-binding domains within the endosomal sorting complexes required for transport (ESCRT) machinery. At the early endosome, the ESCRT-0 complex binds to ubiquitinated receptors and recruits the ESCRT-I complex [15]. Ubiquitinated receptors are then sorted to the limiting membrane of late endosomes where the ESCRT-II complex promotes packaging of receptors into intraluminal vesicles (ILVs) [16, 17]. The ESCRT-III complex polymerizes into spiral filaments on the endosomal membrane to facilitate budding of intraluminal vesicles [18]. The AAA-ATPase Vps4 removes ESCRT-III filaments, which is followed by ILV scission and the formation of multivesicular endosomes (MVEs) [19, 20]. Receptors are then degraded within the lumen of MVEs that fuse to lysosomes.

In contrast, the adaptor protein ALIX binds directly to the G protein-coupled protease-activated receptor-1 (PAR1) independent of receptor ubiquitination and recruits the ESCRT-III complex to facilitate PAR1 sorting into ILVs [4], bypassing the requirement for ubiquitin-binding ESCRT components. ALIX is a multivalent adaptor protein that functions in cytokinesis, viral budding at the plasma membrane and protein sorting at the multivesicular body [21, 22]. ALIX binds directly to a YPX_3_L motif (where X is any amino acid) within the intracellular loop 2 (ICL2) of PAR1 [4]. A bioinformatic search of all known human GPCR sequences identified YPX_3_L motifs in multiple receptors, including P2Y_1_, which harbors a highly conserved YPX_3_L motif [4]. These findings suggest that ALIX may regulate the lysosomal sorting of multiple GPCRs by binding to YPX_3_L sorting motifs but this has not yet been formally tested.

In the present study, we investigated the mechanisms that control P2Y_1_ lysosomal degradation following prolonged ADP stimulation. We demonstrate that agonist-induced P2Y_1_ lysosomal degradation does not require receptor ubiquitination or the ubiquitin-binding ESCRT-0 subunit hepatocyte growth factor regulated tyrosine kinase substrate (HRS) but rather is dependent on ALIX and the YPX_3_L sorting motif present in the ICL2 of P2Y_1_. The P2Y_1_ YPX_3_L mutant internalizes from the plasma membrane following agonist stimulation but failed to sort into the lumen of CD63-positive late endosomes, suggesting that the YPX_3_L motif is required for sorting into late endosomes. The P2Y_1_ YPX_3_L motif is required for interaction with ALIX following ADP stimulation. In addition, siRNA-mediated depletion of endogenous ALIX blocks agonist-promoted P2Y_1_ degradation. These results demonstrate a novel function for ALIX in the lysosomal degradation of P2Y_1_ and support the hypothesis that ALIX regulates the ubiquitin-independent trafficking of GPCRs that harbor YPX_3_L motifs.

## Materials and Methods

### Reagents and antibodies

ADP was purchased from Acros Organics (Fair Lawn, NJ). Leupeptin was obtained from Calbiochem (Billerica, MA). Proteinase K was from Invitrogen (Carlsbad, CA). Triton X-100 was purchased from Thermo Fisher Scientific (Fair Lawn, NJ). Polyclonal anti-FLAG, anti-HA and anti-GFP antibodies were from Rockland Immunochemicals (Pottstown, PA). Monoclonal anti-ALIX, and monoclonal anti-Ub (P4D1) antibodies were obtained from Santa Cruz Biotechnology (Santa Cruz, CA). Monoclonal anti-HA antibody was from Covance (San Diego, CA). Monocolonal anti-early endosomal antigen-1 (EEA1) antibody was obtained from BD Biosciences (East Rutherford, NJ). Monoclonal anti-actin antibody was from Sigma-Aldrich (St. Louis, MO). Monoclonal anti-HRS antibody was from Genetex (Irvine, CA). Monoclonal EGFR antibody (clone LA22) was from Millipore (San Diego, CA). HRP-conjugated goat-anti rabbit and goat-anti mouse antibodies were purchased from Bio-Rad Laboratories (Irvine, CA). Alexa Fluor 488 secondary antibody was from Invitrogen.

### Cell culture and plasmids

HeLa cells were grown and maintained in Dulbecco’s Modified Eagle Medium (Mediatech, Oceanside, CA) supplemented with 10% fetal bovine serum (Thermo Fisher Scientific) and 1% penicillin/streptomycin (Thermo Fisher Scientific). The full-length human N-terminal HA-tagged P2Y_1_ cDNA cloned into the mammalian expression vector pcDNA3.1 was provided by Dr. Robert Nicholas (University of North Carolina, Chapel Hill, NC). The HA-P2Y_1_ 3KR mutant cloned into pcDNA3.1 was generated by site directed mutagenesis as described previously [23]. The full-length N-terminal FLAG-tagged P2Y_1_ was generated by insertion of the P2Y_1_ open reading frame into a FLAG pcDNA3.1 vector and verified by dideoxy sequencing (Retrogen, San Diego, CA). The FLAG-P2Y_1_ Y155A mutant was generated by mutating residues encoding the critical tyrosine t65g, a66c, and c67a using QuikChange site-directed mutagenesis (Agilent Technologies, La Jolla, CA) and verified by dideoxy sequencing (Retrogen). The Vps4 WT and E228Q fused to green fluorescence protein (GFP) were generously provided by Dr. Adriano Marchese (Loyola University, Maywood, IL). The CD63 fused to red fluorescence protein (RFP) plasmid was obtained from Dr. Robert Parton (University of Queensland, Queensland, Australia) [24].

### siRNA and cDNA transfections

HeLa cells were transfected with plasmid DNA using 1 mg/ml polyethyleninmine (PEI, Polysciences Inc, Warrington, PA) at a 8:1 ratio (8μl PEI:1μg plasmid). siRNA transfection was performed using Oligofectamine (Invitrogen) per the manufacturer’s instructions. All single siRNAs were purchased from Qiagen or Invitrogen: non-specific: 5’-GGCTACGTCCAGGAGCGCACC-3'; ALIX #1: 5’-AAGTACCTCAGTCTATATTGA-3’; ALIX #3: 5-AATCGAGACGCTCCTGAGATA-3’; HRS: 5’-CGACAAGAACCCACACGUC-3’.

### Immunoprecipitation assays

HeLa cells were grown in 6-well plates (2.0 x 10^5^ cells/well) overnight at 37°C, then transfected with either HA-P2Y_1_ or FLAG-P2Y_1_ plasmids. Cells were incubated for 48 h, washed, then stimulated for the indicated times with 10 μM ADP (Acros Organics). P2Y_1_ ubiquitination was determined as follows: Cells were washed, then lysed with RIPA buffer (5 mM EDTA, 50 mM Tris-HCl pH 8.0, 0.5% (w/v) Na Deoxycholate, 1% (v/v) NP-40, 0.1% (w/v), 1% SDS) supplemented with 50 mM β-glycerophosphate, 10 μg/ml leupeptin, aprotinin, trypsin protease inhibitor, pepstatin, 100 μg/ml benzamide and 3 mg/ml NEM (Calbiochem) as described previously [23]. P2Y_1_ co-immunoprecipitations were performed as follows: Cells were washed, then lysed with NP-40 buffer (0.5% (v/v) NP40, 20 mM Tris-HCl pH 7.4, 150 mM NaCl) supplemented with 10 μg/ml leupeptin, aprotinin, trypsin protease inhibitor, pepstatin, 100 μg/ml benzamide and 3 mg/ml NEM. Lysates were sonicated for 10 s at 10% amplitude (Branson Model 450 sonifier), then clarified by centrifugation at 14,000 rpm for 30 min at 4°C. Protein concentrations were determined by bicinchoninic acid (BCA) analysis (Thermo Fisher Scientific) and equivalent amounts of cell lysates were incubated with protein-A sepharose beads (GE Healthcare) and the indicated antibodies overnight at 4°C. Beads were washed and resuspended in 2X Laemmli sample buffer. Samples were then resolved by SDS-PAGE and transferred to PVDF membrane, then analyzed by immunoblotting and chemiluminescence. Films were scanned and quantified by densitometry using ImageJ software (NIH, Bethesda, MD).

### Degradation assays

For P2Y_1_ degradation assays, HeLa cells transiently transfected with either HA-P2Y_1_ or FLAG-P2Y_1_ plasmids were plated in 12-well plates (0.75 x 10^5^ cells/well) and grown overnight at 37°C. For siRNA transfection experiments, cells were first transfected with plasmids, incubated overnight, then transfected with siRNA. For EGFR degradation assays, HeLa cells were plated in 12-well plates (0.75 x 10^5^ cells/well). After 48 h, cells were washed, then incubated with or without 10 μM ADP (P2Y_1_ assays) or 10 nM EGF (EGFR assays) at 37°C for the indicated times. Cells were then placed on ice, washed with phosphate buffered saline (PBS), and lysed in Triton X-100 lysis buffer (50 mM Tris-HCl, pH 7.4, 100 mM NaCl, 5 mM EDTA, 50 mM NaF, 10 mM NaPP, and 1% (v/v) Triton X-100) supplemented with protease inhibitors as described above. Cell lysates were collected and sonicated for 10 s at 10% amplitude, and protein concentrations were determined by BCA assay (Thermo Fisher Scientific). Equivalent amounts of lysates were then resolved by SDS-PAGE, transferred to PVDF membrane and analyzed by immunoblotting and chemiluminescence. Immunoblots were quantified by densitometry using ImageJ software.

### Cell surface enzyme linked immunosorbant assay (ELISA)

HeLa cells transiently transfected with the indicated plasmids were plated in 24-well plates (0.5 x 10^5^ cells/well) and incubated overnight. For siRNA experiments, cells were transfected with plasmid, incubated overnight and then transfected with siRNA. Cells were stimulated with 10 μM ADP for the indicated times, then washed with ice-cold PBS. Cells were then fixed with 4% paraformaldehyde (PFA), washed with PBS and incubated with anti-FLAG antibody for 1 h. Cells were washed and incubated with HRP-conjugated secondary antibody for 1 h at room temperature, then developed using 1-Step ABTS (2,2'-Azinobis [3-ethylbenzothiazoline-6-sulfonic acid]-diammonium salt) (Thermo Fisher). The amount of surface P2Y_1_ was quantified by determining the absorbance of an aliquot at 405 nm using a Spectramax Plus (Molecular Dynamics) spectrophotometer.

### Immunofluorescence confocal microscopy

HeLa cells were plated on coverslips in 12-well plates (0.75 x 10^5^ cells/well) and incubated overnight at 37°C. Cells were transfected with the indicated plasmids, then incubated for 48 h. For experiments examining the colocalization of P2Y_1_ with RFP-CD63, cells were pre-treated with 2 mM leupeptin for 1 h at 37°C to inhibit lysosomal degradation. Cells were washed then labeled with anti-FLAG antibody at 4°C for 1 h, under these conditions only the cell surface receptor cohort bound antibody. Cells were washed and stimulated with 10 μM ADP for the indicated times. Cells were fixed in 4% PFA, permeabilized in 100% methanol and treated with the indicated primary and secondary antibodies. Coverslips were mounted using FluorSave Reagent (EMD Millipore). Images were acquired with a spinning-disk confocal system (Olympus) configured with a microscope (IX81, Olympus) fitted with a PlanApo 60x oil objective (1.4 NA, Olympus) and a digital camera (ORCA-ER, Hamamatsu Photonics). Fluorescent images of 0.28μm-thick X-Y sections were acquired sequentially at room temperature using Slidebook 5.0 software (Intelligent Imaging Innovations). Pearson’s correlation coefficients for quantifying colocalization were calculated from at least six independent cells from multiple experiments using Slidebook 5.0.

### Proteinase-K Protection Assay

The proteinase-K protection assay was performed as described [25, 26]. HeLa cells were plated in six-well culture dishes (4.0 X 10^5^ cells/well), grown overnight at 37°C and transfected with FLAG-P2Y_1_ wildtype (WT) or FLAG-P2Y_1_ Y155A, as described above. Cells were incubated with or without 10 μM ADP and 1 mM Leupeptin (Calbiochem). Cells were placed on ice and incubated for 5 min with PBS, harvested and gently permeabilized using 6.5 μg/ml digitonin (Thermo Fisher Scientific). Membranes were collected by centrifugation and resuspended in buffer (100 mM K_2_HPO_4_/KH_2_PO_4_, 5 mM MgCl_2_, 250 mM sucrose). Membranes were then divided into three aliquots either left untreated, treated with 2.5 ng/ml proteinase-K, or treated with proteinase-K supplemented with 0.1% Triton-X 100 for 10 min at room temperature. After treatments, samples are diluted with 100 μl 2X SDS sample buffer containing 20 mM PMSF and analyzed by immunoblotting as described above.

### Statistical analysis

Data were analyzed using Prism software (version 4.0, GraphPad Software, San Diego, CA). Statistical analysis was determined, as indicated, by performing either Student’s *t*-test, one-way or two-way analysis of variance (ANOVA).

## Results

### P2Y_1_ ubiquitination is not required for degradation

**Fig 1:**
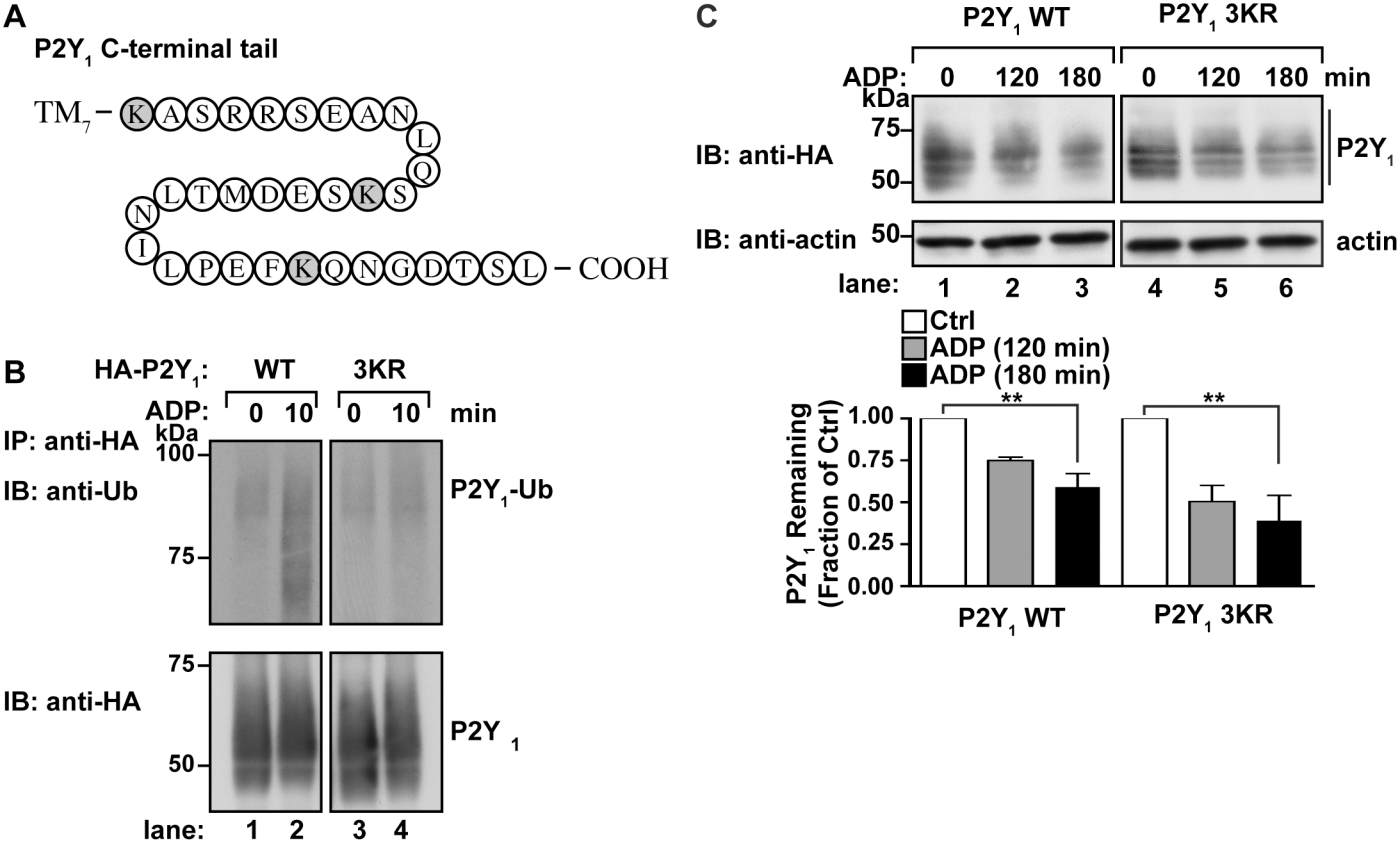
ADP-stimulated ubiquitination of P2Y_1_ is not required for degradation. (A) A diagram of the P2Y_1_ C-terminal tail residues. Lysine residues (K) that represent potential ubiquitination sites are shaded in grey. (B) HeLa cells transfected with HA-P2Y_1_ WT or the HA-P2Y_1_ 3KR mutant were stimulated with 10 μM ADP, lysed and equivalent amounts of cell lysates were immunoprecipitated and immunoblotted with anti-HA antibody for P2Y_1_ and anti-ubiquitin antibody. (C) HeLa cells transfected with HA-P2Y_1_ WT or the HA-P2Y_1_ 3KR mutant were stimulated with 10 μM ADP for the indicated times, lysed and equivalent amounts of cell lysate immunoblotted to with anti-HA and anti-actin. The amount of P2Y_1_ was quantified using densitometry, and the data (mean ± S.D., n = 3) are representative of three separate experiments, expressed as the fraction of receptor levels in unstimulated cells and were analyzed by ANOVA (**, *P* < 0.01).

Previous studies have shown that the P2Y_1_ receptor is sorted to lysosomes and degraded following stimulation with ADP in 1321N1 human astrocytoma cells [14], however the mechanisms that control P2Y_1_ degradation are unclear. Ubiquitin conjugated to lysine (K) residues localized within the intracellular loops or C-terminal terminal tail serve as a lysosomal sorting signal for many GPCRs, including protease-activated receptor-2 (PAR2) [27], CXCR4 [28], and the β2-adrenergic receptor [29]. In contrast, a recent study demonstrated that P2Y_1_ ubiquitination mediates receptor signaling to the p38 mitogen-activated protein kinase [23], however the function of P2Y_1_ ubiquitination in lysosomal sorting is not known. There are three lysine residues present within the C-terminal tail of human P2Y_1_ receptor (Fig 1A), that have been shown to serve as sites for P2Y_1_ ubiquitination [23]. To confirm that these residues are required for ubiquitination of P2Y_1_, we expressed a mutant of P2Y_1_ receptor in which the C-terminal tail lysine residues were mutated to arginine (R) that is designated P2Y_1_ 3KR. HeLa cells transiently transfected with HA-P2Y_1_ wild type (WT) or the HA-P2Y_1_ 3KR mutant were stimulated with 10 μM ADP for 10 min. Cells were lysed, immunoprecipitated with anti-HA antibodies, and ubiquitination of P2Y_1_ was assessed by immunoblotting. In the absence of agonist, ubiquitination of P2Y_1_ WT or 3KR was minimally detected (Fig 1B, lanes 1 and 3). However, ADP stimulation of P2Y_1_ WT for 10 min induced a marked increase in receptor ubiquitination (Fig 1B, lane 2). In contrast, ubiquitination of P2Y_1_ 3KR mutant is not detectable following stimulation with ADP (Fig 1B, lane 4). These results suggest that activation of the P2Y_1_ receptor induces ubiquitination at the C-terminal tail lysine residues.

We next investigated whether agonist-induced ubiquitination of the P2Y_1_ receptor is required for lysosomal sorting and degradation. HeLa cells expressing HA-P2Y_1_ WT or HA-P2Y_1_ 3KR were stimulated with 10 μM ADP for the indicated times and the amount of P2Y_1_ protein remaining in cell lysates was determined by immunoblotting. Wild-type P2Y_1_ displayed ~50% degradation following stimulation with ADP for 180 min (Fig 1C, lanes 1–3). Interestingly, ADP-activated P2Y_1_ 3KR mutant also exhibited ~50% degradation at 180 min that was comparable to P2Y_1_ WT (Fig 1C, lanes 4–6). These findings suggest that P2Y_1_ degradation induced by prolonged agonist stimulation does not require receptor ubiquitination.

### P2Y_1_ degradation is independent of HRS and Vps4

**Fig 2:**
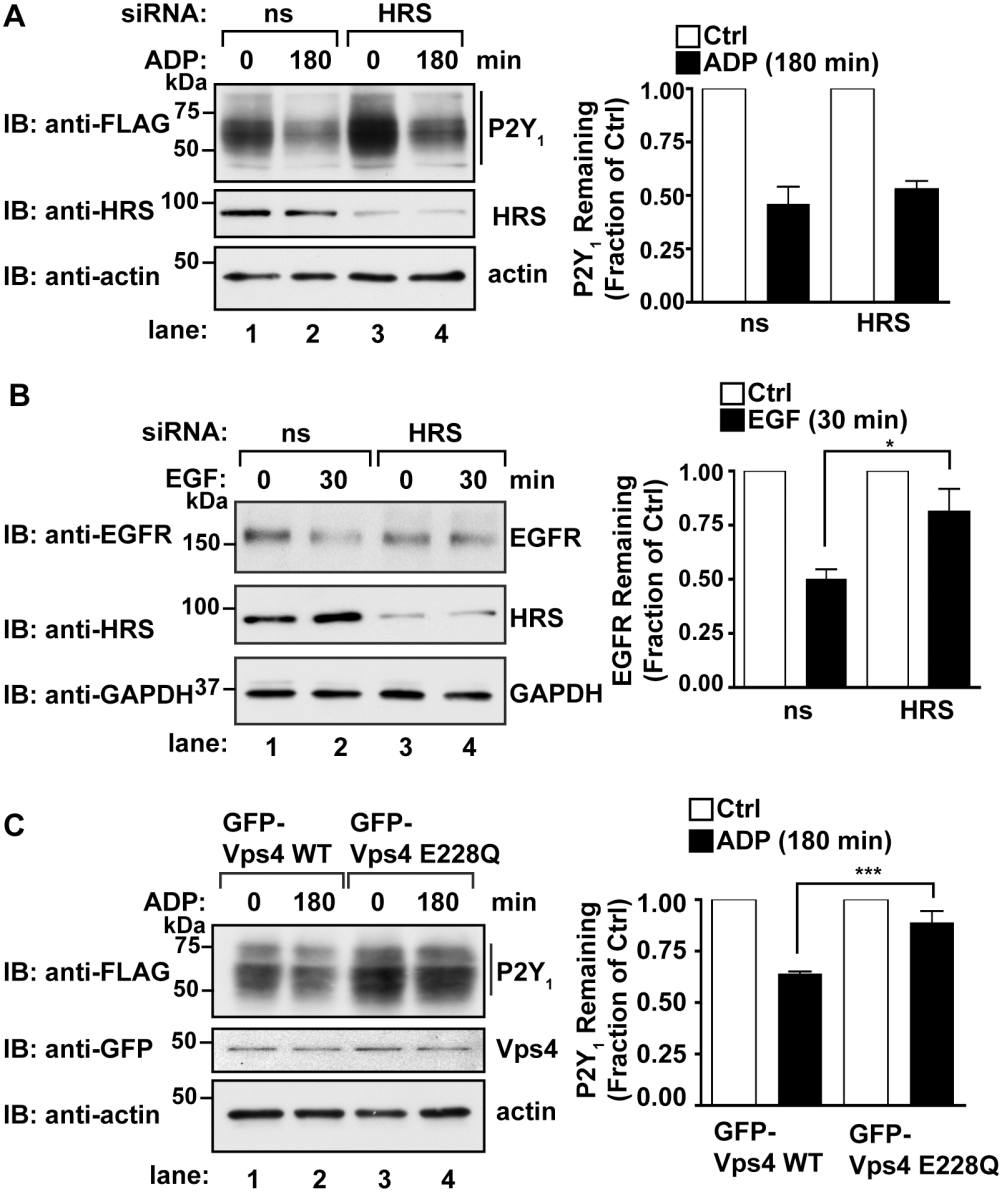
P2Y_1_ degradation is independent of HRS but requires Vps4. (A) HeLa cells co-transfected with FLAG-P2Y_1_ WT and either non-specific (ns) or HRS siRNAs were stimulated with 10 μM ADP, lysed and equivalent amounts of lysate were immunoblotted with anti-FLAG, anti-HRS and anti-actin antibodies. The amount of HRS expressed in HRS knockdown cells was quantified using densitometry and compared to levels in non-specific siRNA-treated cells (23.0 ± 0.08%, n = 3). The amount of P2Y_1_ was quantified using densitometry. The data (mean ± S.D.) represent three independent experiments, are expressed as the fraction of receptor levels in unstimulated cells, and the affect of HRS depletion on P2Y_1_ degradation was assessed using Student’s *t* test. (B) HeLa cells were transfected with non-specific (ns) or HRS siRNAs and stimulated with or without 10 nM EGF. Equivalent amounts of lysates were immunoblotted with anti-EGFR, anti-HRS or anti-GAPDH antibodies. The amount of HRS expressed in HRS knockdown cells was quantified using densitometry and compared to levels in non-specific siRNA-treated cells (22.2 ± 0.06%, n = 3). The amount of endogenous EGFR was quantified using densitometry. The data (mean ± S.D.) represent three independent experiments, and are expressed as the fraction of receptor levels in unstimulated cells. The affect of HRS depletion on EGFR degradation was assessed using Student’s *t* test (*, *p <* 0.05). (C) HeLa cells co-transfected with FLAG-P2Y_1_ WT and either GFP-Vps4 WT or GFP-Vps4 E228Q were stimulated with 10 μM ADP, lysed and equivalent amounts of lysate were immunoblotted with anti-FLAG, anti-GFP and anti-actin antibodies. The amount of P2Y_1_ remaining in cell lysates was quantified by densitometry. The data (mean ± S.D., n = 3) are expressed as the fraction of P2Y_1_ levels in unstimulated cells and was analyzed by Student’s *t*-test (***, *P* < 0.001).

To test whether ubiquitin-binding ESCRTs mediate P2Y_1_ lysosomal sorting, siRNAs were used to deplete cells of endogenous HRS, a ubiquitin-binding component of the ESCRT-0 complex [30]. HeLa cells were co-transfected with FLAG-P2Y_1_ and either non-specific siRNA or siRNA specifically targeting HRS [31]. Cells were then stimulated with ADP and the amount of P2Y_1_ protein remaining was determined by immunoblotting. In non-specific siRNA treated cells, ~50% of P2Y_1_ receptor was degraded following agonist activation compared to untreated control cells (Fig 2A, lanes 1 and 2). Compared to unstimulated control cells, ADP-stimulated P2Y_1_ also displayed a substantial ~50% degradation in cells depleted of HRS (Fig 2A, lane 4). To confirm HRS depletion-effects on receptor degradation, we examined the lysosomal sorting of epidermal growth factor receptor (EGFR), a receptor that requires ubiquitination and HRS for degradation [32]. HeLa cells expressing endogenous EGFR were transfected with non-specific or HRS siRNA, then stimulated with 10 nM EGF for 30 min. In cells transfected with non-specific siRNA, EGF stimulation resulted in ~50% loss of EGFR compared to untreated cells (Fig 2B, lanes 1 and 2). The efficiency of HRS knockdown was comparable to HRS siRNA-treated samples co-transfected with P2Y_1_. However, only ~15% of EGFR was degraded following stimulation of cells depleted of endogenous HRS (Fig 2B, lanes 3 and 4). These results are consistent with previous studies demonstrating that HRS is required for EGFR lysosomal sorting and degradation [33]. Together, these findings suggest that the degradation of activated P2Y_1_ is mediated by an ubiquitin-independent mechanism that does not require HRS, a ubiquitin-binding component of ESCRT-0.

Vps4 is expressed as an oligomer of ATPase subunits, and Vps4-mediated intraluminal vesicle scission is disrupted by overexpression of a dominant-negative mutant, Vps4 E228Q that lacks ATPase activity [34]. To examine the function of Vps4 in the lysosomal sorting and degradation of P2Y_1_, the Vps4 E228Q mutant was co-expressed with FLAG-P2Y_1_ and agonist-induced degradation of P2Y_1_ was assessed. In control cells transfected with Vps4 wildtype, P2Y_1_ was degraded by ~50% after stimulation with ADP (Fig 2C, lane 1 and 2). However, in cells expressing the Vps4-E228Q mutant there was significantly less degradation, only ~15% after ADP stimulation (Fig 2C, lane 3 and 4), These results suggest that agonist-induced lysosomal degradation of P2Y_1_ occurs through a Vps4-dependent pathway independent of HRS.

### The YPX_3_L motif is required for agonist-induced P2Y_1_ degradation

**Fig 3:**
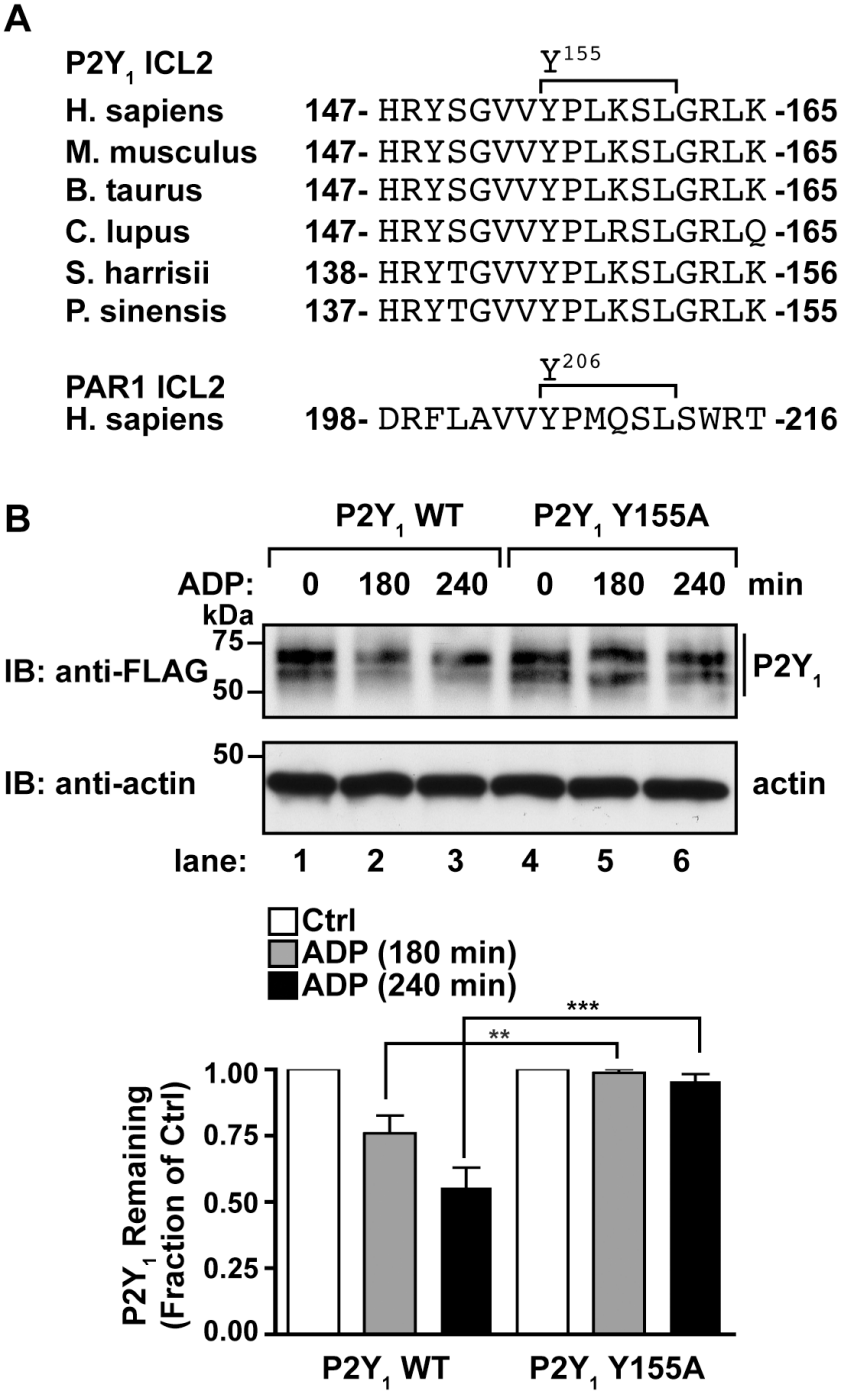
P2Y_1_ is targeted for degradation by a YPX_3_L motif. (A) An alignment of P2Y_1_ intracellular loop 2 (ICL2) sequences from various species. The PAR1 ICL2 sequence is provided for comparison. (B) HeLa cells transfected with either FLAG-P2Y_1_ WT or the FLAG-P2Y_1_ Y155A mutant were stimulated with 10 μM ADP for the indicated times and cell lysates were analyzed by immunoblot. The amount of P2Y_1_ remaining (mean ± S.D.) is expressed as a fraction of P2Y_1_ relative to receptor levels in untreated cells. Mutation of the P2Y_1_ YPX_3_L motif significantly inhibited receptor degradation, as analyzed by two-way ANOVA (**, *P* < 0.01; ***, *P* < 0.001; n = 3).

P2Y_1_ has a highly conserved Y^155^PLKSL^160^ sequence within its ICL2 that conforms to a YPX_3_L motif, similar to that found in PAR1 (Fig 3A) [4]. Mutation of the critical tyrosine Y^206^ residue to alanine present with the PAR1 YPX_3_L motif is sufficient to block agonist-induced PAR1 lysosomal degradation [4]. To test whether the YPX_3_L motif of P2Y_1_ is required for lysosomal degradation, the tyrosine-155 (Y^155^) residue was mutated to alanine to generate the P2Y_1_ Y155A mutant receptor (Fig 3A). HeLa cells transiently transfected with FLAG-P2Y_1_ WT or the FLAG-P2Y_1_ Y155A mutant were stimulated with 10 μM ADP for the indicated times and degradation of P2Y_1_ was assessed by immunoblotting. Wild-type P2Y_1_ was degraded by ~50% after 240 min incubation with ADP compared to untreated control cells (Fig 3B, lanes 1–3). In contrast, agonist stimulation of the P2Y_1_ Y155A mutant failed to promote receptor degradation, and only ~5% of receptor was degraded after 240 min (Fig 3B, lanes 4–6). These results suggest that the P2Y_1_ YPX_3_L motif is required for agonist-induced receptor degradation.

### The P2Y_1_ YPX_3_L motif is required for lysosomal sorting

**Fig 4:**
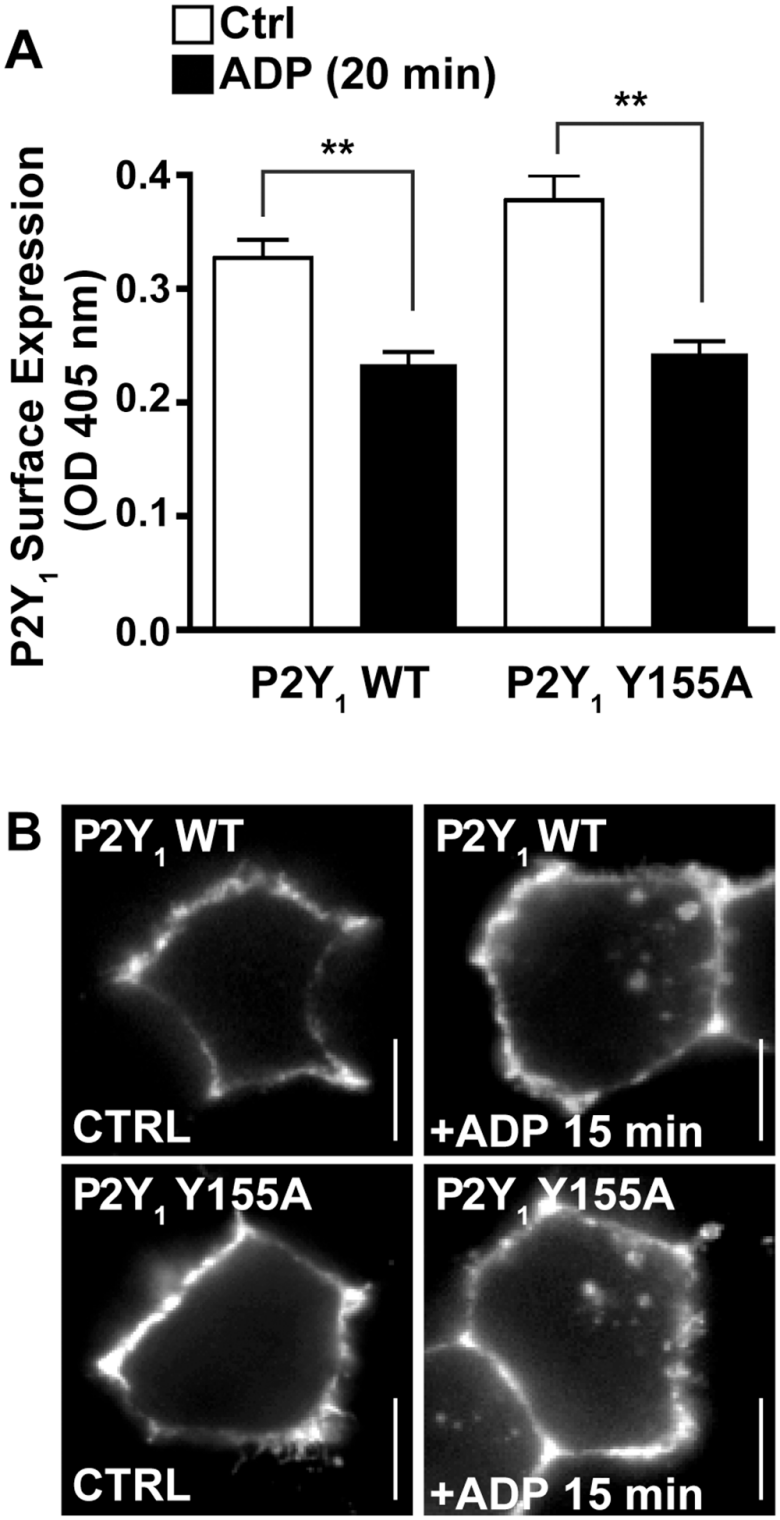
Mutation of the P2Y_1_ YPX_3_L motif does not block receptor internalization. (A) HeLa cells transfected with FLAG-P2Y_1_ WT or FLAG-P2Y_1_ Y155A were surface-labeled with anti-FLAG antibody, then stimulated with or without 10 μM ADP. Control (Ctrl) cells were surface labeled on ice as a measure of initial surface P2Y_1_. Cells were then fixed and labeled with secondary antibody conjugated to HRP. The amount of P2Y_1_ remaining on the cell surface was quantified by ELISA. The data (mean ± S.D.) are expressed as the absorbance (OD) at 405nm and are representative of three independent experiments. The extent of P2Y_1_ internalization is significant, as analyzed using Student’s *t-*test (**, *P* < 0.01). (B) HeLa cells transfected with FLAG-P2Y_1_ WT or FLAG-P2Y_1_ Y155A were surface labeled with anti-FLAG antibody, then stimulated with 10 μM ADP for 15 min. Cells were fixed, labeled with fluorescent secondary antibodies, and the internalization of P2Y_1_ was visualized by confocal microscopy. The images shown are representative of many cells examined in three independent experiments. Scale bars = 10 μm.

We next examined whether the P2Y_1_ YPX_3_L motif is important for receptor internalization. HeLa cells expressing FLAG-P2Y_1_ WT or the FLAG-P2Y_1_ Y155A mutant were incubated with anti-FLAG antibody at 4°C to label the cell surface receptor cohort prior to treatment with or without ADP for 20 min at 37°C. After stimulation, the amount of P2Y_1_ remaining at the cell surface was measured using ELISA. P2Y_1_ WT and the P2Y_1_ Y155A mutant showed equal surface expression and exhibited a similar loss in cell surface expression following 20 min of stimulation with ADP (Fig 4A), suggesting that both receptors internalize comparably. Agonist-induced internalization of P2Y_1_ WT and Y155A mutant was confirmed by confocal immunofluorescence microscopy. HeLa cells transiently transfected with FLAG-P2Y_1_ WT or FLAG-P2Y_1_ Y155A were incubated with anti-FLAG antibody at 4°C to label the surface cohort, then stimulated with ADP for 15 min at 37°C. Cells were fixed and internalized P2Y_1_ was detected by immunostaining. Both P2Y_1_ WT and the P2Y_1_ Y155A mutant localized to the cell surface in control cells. However, after ADP stimulation both wildtype and mutant P2Y_1_ redistributed from the cell surface and localized to internal punctae (Fig 4B). These findings suggest that the YPX_3_L motif of P2Y_1_ does not regulate receptor internalization induced by agonist activation.

**Fig 5:**
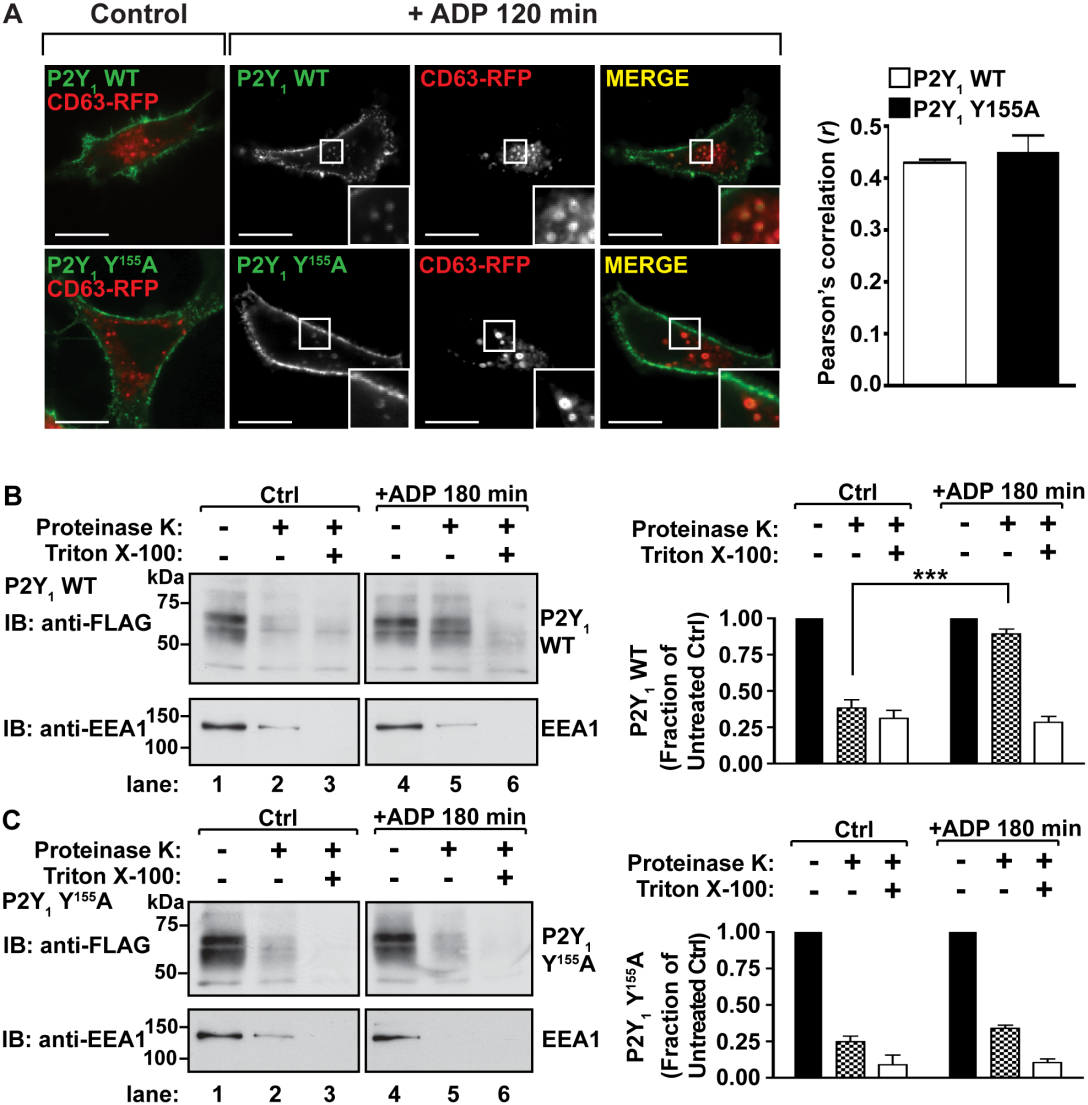
The P2Y_1_ YPX_3_L motif mediates sorting of P2Y_1_ into the lumen of multivesicular endosomes. (A) HeLa cells co-transfected with RFP-CD63 and either FLAG-P2Y_1_ WT or FLAG-P2Y_1_ Y155A were surface-labeled with anti-FLAG antibody, then stimulated for 120 min with 10 μM ADP. Cells were fixed and immunostained. The internalization of P2Y_1_ to CD63-positive endosomes was visualized by confocal microscopy and the extent of co-localization between P2Y_1_ and CD63 was quantified by Pearson’s correlation. The data (mean ± S.D.) are representative of three independent experiments. (B and C) HeLa cells were transfected with FLAG-P2Y_1_ WT (B) or FLAG-P2Y_1_ Y155A (C) and stimulated for 180 min. Cells were gently permeabilized with digitonin, then separated into three fractions that were treated with either H_2_O (Ctrl), Proteinase-K, or Proteinase-K with Triton X-100. The amount of receptor within each fraction was determined by immunoblot. The data shown (mean ± S.D.) are expressed as the fraction of P2Y_1_ compared to receptor detected in the untreated fraction and are representative of three independent experiments. P2Y_1_ immunoblots were quantified by densitometry and analyzed by two-way ANOVA (***, *P* < 0.001).

To investigate whether the YPX_3_L motif of P2Y_1_ mediates sorting to late endosomes, confocal immunofluorescence microscopy was used to determine whether P2Y_1_ wild-type or Y155A mutant co-localized with CD63, a protein that resides at the limiting membrane of intraluminal vesicles of late endosomes/multivesicular bodies [35]. HeLa cells transiently transfected with FLAG-P2Y_1_ WT or FLAG-P2Y_1_ Y155A together with CD63-RFP were incubated with anti-FLAG antibodies to label the cell surface receptor cohort, then stimulated for 120 min with ADP at 37°C. In control cells, both P2Y_1_ WT and Y155A localized primarily at the plasma membrane, whereas CD63-RFP was present in internal punctae (Fig 5A). After stimulation with ADP for 120 min, both P2Y_1_ WT and Y155A mutant sorted to CD63-positive endosomes (Fig 5A). Pearson’s correlation coefficients between P2Y_1_ WT or Y155A and CD63 were calculated and revealed that the extent of colocalization between CD63 and P2Y_1_ WT and P2Y_1_ Y155A was comparable (Fig 5A). These data suggest that the P2Y_1_ YPX_3_L motif is not required for receptor transport to late endosomes/multivesicular bodies.

The PAR1 YPX_3_L motif is required for receptor sorting into intraluminal vesicles that bud inward from the limiting membrane of multivesicular bodies [4]. Thus, to examine whether the P2Y_1_ YPX_3_L motif also mediates receptor sorting into the lumen of multivesicular bodies we used a proteinase-K protection assay [26]. HeLa cells transiently transfected with FLAG-P2Y_1_ WT or Y155A mutant were pretreated with 2 mM leupeptin to inhibit lysosomal degradation and incubated with or without ADP for 180 min. The cells were briefly treated with digitonin to permeabilize the plasma membrane, while leaving endosomal membranes intact. Equivalent amounts of permeabilized cells were then treated with H_2_O (Ctrl), Proteinase-K alone, or Proteinase-K in 1% Triton X-100 that permeabilizes the plasma membrane and endosomal membranes. P2Y_1_ receptors that are sorted into the lumen of multivesicular endosomes are protected from proteinase-K degradation, whereas early endosomal antigen-1 (EEA1), which localizes to the limiting membrane of endosomes, is sensitive to proteolytic cleavage by proteinase K. The extent of proteinase-K-mediated protein degradation was assessed by immunoblotting. In unstimulated cells, proteinase-K treatment degraded ~70% of P2Y_1_ WT receptors, compared to H_2_0 –treated controls (Fig 5B, lanes 1 and 2). Similarly, treatment with proteinase-K degraded EEA1, confirming digitonin-mediated permeabilization of the plasma membrane. Treatment with proteinase-K and Triton-X100 detergent resulted in a 75% degradation of P2Y_1_ (Fig 5B, lane 3). These results are consistent with P2Y_1_ expression at the cell surface and on limiting membranes of endosomes with only a small fraction of P2Y_1_ sorted into protective endosomal compartments under basal conditions. In contrast cells treated with ADP for 180 min, proteinase-K treatment degraded only ~20% of P2Y_1_ WT, compared to H_2_O-treated control (Fig 5B, lanes 4 and 5). However, treatment with proteinase-K and Triton X-100 degraded ~75% of P2Y_1_ WT receptors (Fig 5B, lane 6), indicating that wild-type P2Y_1_ is sorted into protective endosomal compartments and is only accessible to proteinase K after permeabilization of all membranes with Triton X-100. Similarly, in unstimulated cells, proteinase-K degrades ~75% of the P2Y_1_ Y155A mutant receptors (Fig 5C, lane 2). However, P2Y_1_ Y155A mutant stimulated with ADP is also sensitive to proteinase K, resulting in ~75% receptor degradation (Fig 5C, lane 5), suggesting that P2Y_1_ Y155A is not sorted into protective endosomal compartments. These findings demonstrate that the P2Y_1_ YPX_3_L motif is required for the sorting of P2Y_1_ into the lumen of multivesicular bodies.

### ALIX regulates P2Y_1_ degradation

**Fig 6:**
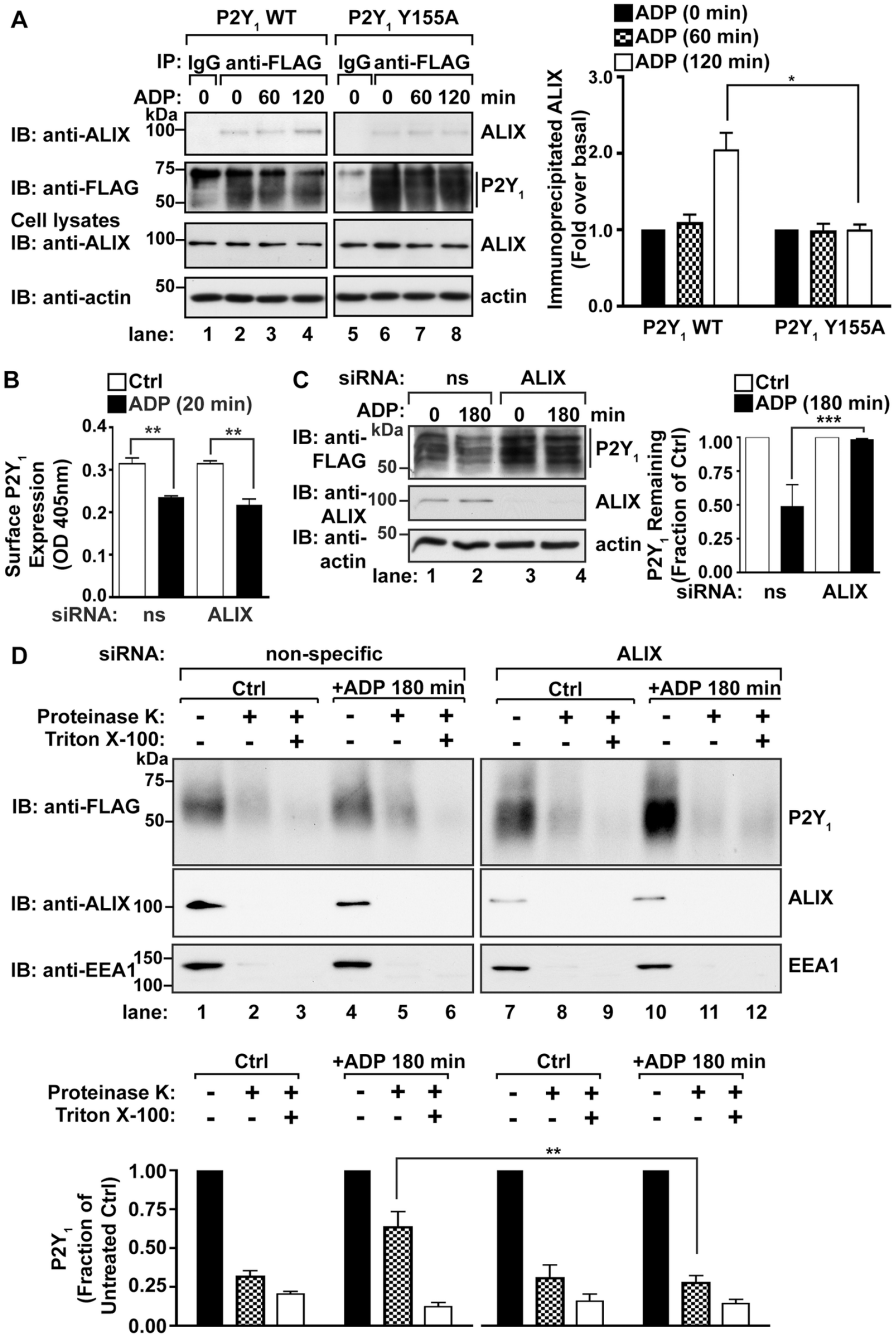
ALIX interacts with P2Y_1_ and mediates P2Y_1_ degradation. (A) HeLa cells transfected with FLAG-P2Y_1_ WT or FLAG-P2Y_1_ Y155A were stimulated with 10 μM ADP for the indicated times. Cell lysates were immunoprecipitated with anti-FLAG antibody and precipitates were analyzed for the presence of endogenous ALIX by immunoblot. Co-immunoprecipitated ALIX was quantified using densitometry. The data (mean ± S.D.) are representative of three independent experiments, and were analyzed by Student’s *t* test (*, *P* < 0.05). (B) Depletion of endogenous ALIX does not inhibit P2Y_1_ internalization. HeLa cells cotransfected with FLAG-P2Y_1_ WT and either non-specific (ns) or ALIX siRNAs were surface labeled with anti-FLAG antibody prior to stimulation with 10 μM ADP for 20 min. The amount of surface P2Y_1_ remaining was quantified by ELISA. The data (mean ± S.D.) are expressed as the absorbance (OD) at 405nm, and are representative of three independent experiments. The extent of P2Y_1_ internalization was significant, as analyzed using Student’s *t-*test (**, *P* < 0.01) analyzed using Student’s *t*-test (**, *P* < 0.01, n = 3). (C) HeLa cells co-transfected with FLAG-P2Y_1_ WT and either non-specific (ns) or ALIX siRNAs were stimulated with 10 μM ADP for 180 min. The amount of P2Y_1_ in cell lysates was analyzed by immunoblot. P2Y_1_ levels were quantified and the data shown (mean ± S.D.) are expressed as the fraction of P2Y_1_ remaining following agonist stimulation and are representative of three experiments. ALIX depletion significantly inhibited P2Y_1_ degradation, as analyzed by two-way ANOVA (***, *P* < 0.001). (D) HeLa cells co-transfected with FLAG-P2Y_1_ WT and either non-specific or ALIX siRNAs were stimulated with 10 μM ADP for 180 min. Cells were gently permeabilized with digitonin, then separated into three fractions that were treated with either H_2_O (Ctrl), Proteinase-K, or Proteinase-K with Triton X-100. The amount of receptor within each fraction was determined by immunoblot. The data shown (mean ± S.D.) are expressed as the fraction of P2Y_1_ compared to receptor detected in the untreated fraction and are representative of three independent experiments. P2Y_1_ immunoblots were quantified by densitometry and analyzed by two-way ANOVA (**, *P* < 0.01).

We next investigated whether the YPX_3_L binding protein ALIX interacts with P2Y_1_ following agonist stimulation using co-immunoprecipitation. HeLa cells transiently transfected with FLAG-P2Y_1_ WT or the FLAG-P2Y_1_ Y155A mutant were stimulated with ADP for the indicated times. Cell lysates were immunoprecipitated with anti-FLAG antibody to purify P2Y_1_, and precipitates were analyzed by immunoblotting for the co-precipitation of endogenous ALIX. Immunoprecipitation of P2Y_1_ WT from unstimulated cells co-precipitates a small amount of ALIX (Fig 6A, lane 2), however ALIX co-precipitation with P2Y_1_ WT is increased following 120 min of ADP stimulation (Fig 6A, lane 4). These results indicate that stimulation of P2Y_1_ enhances interaction with ALIX. Similar to P2Y_1_ WT, the P2Y_1_ Y155A mutant coprecipitates a very small amount of ALIX from unstimulated cells (Fig 6A, lane 6). In contrast, co-precipitation of ALIX with the P2Y_1_ Y155A mutant was not enhanced following ADP stimulation (Fig 6A, lanes 7 and 8), suggesting that the P2Y_1_ YPX_3_L motif is required for interaction with ALIX. These findings demonstrate that ALIX interacts with stimulated P2Y_1_ and ALIX co-precipitation with P2Y_1_ requires the YPX_3_L motif.

**Fig 7:**
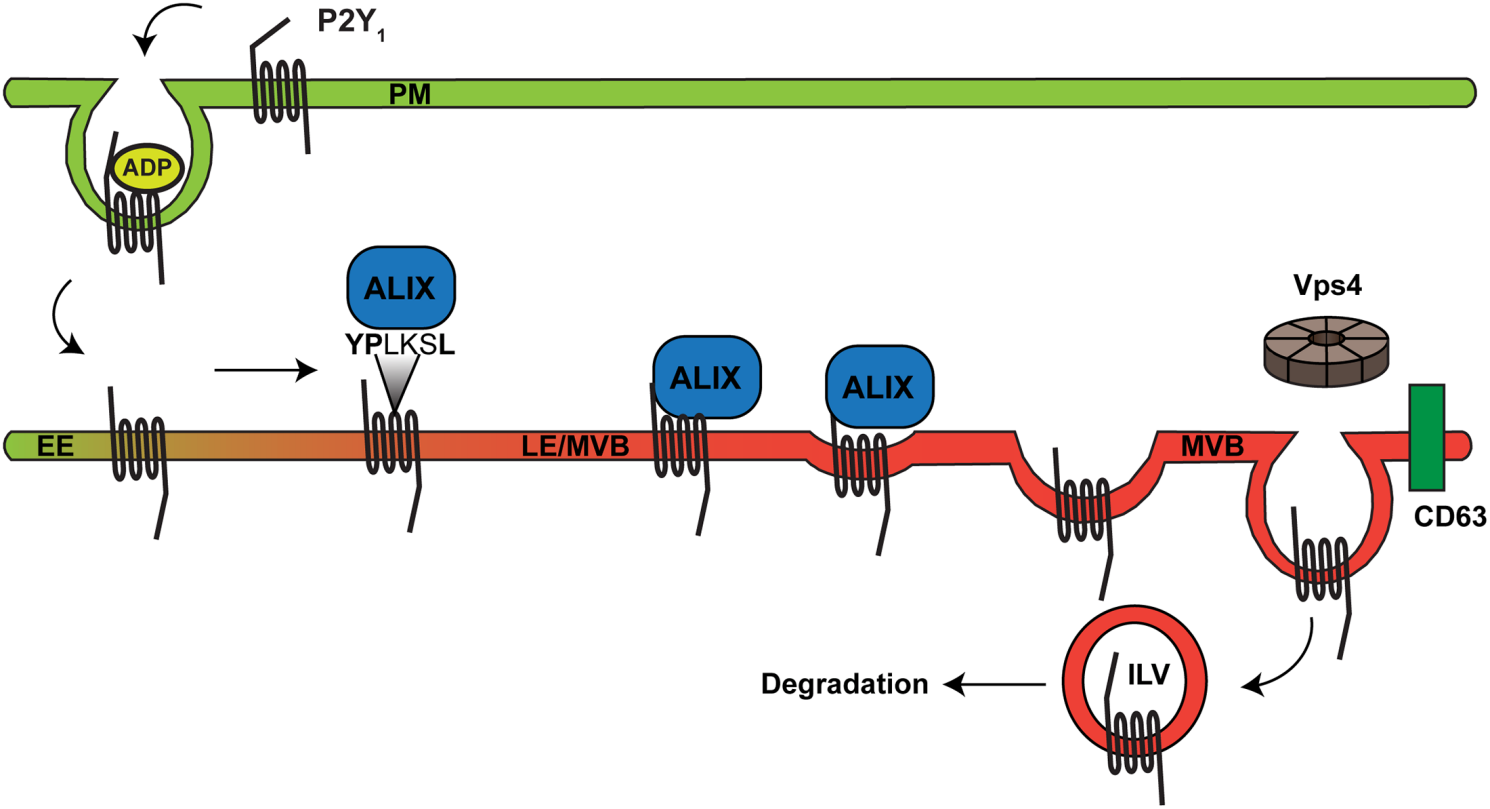
ALIX mediates the sorting of P2Y_1_ at the late endosome/MVB. Similar to other GPCRs, the P2Y_1_ receptor resides primarily on the cell surface in the absence of agonist stimulation. After ADP stimulation, P2Y_1_ is internalized from the plasma membrane to endocytic vesicles and then sorted to CD63-positive late endosomes and degraded in a Vps4-dependent manner following prolonged stimulation. ALIX interacts with a conserved YPX_3_L motif within the ICL2 of P2Y_1_, and facilitates sorting of the receptor at late endosomes into intraluminal vesicles (ILV) of the multivesicular bodies (MVB), where the receptor is eventually degraded.

We next examined whether ALIX mediates P2Y_1_ internalization and lysosomal degradation. To investigate whether ALIX regulates P2Y_1_ internalization, HeLa cells co-transfected with FLAG-P2Y_1_ WT and non-specific (ns) or ALIX siRNAs were incubated with anti-FLAG antibody at 4°C to label the surface cohort of receptors. Cells were incubated with or without ADP at 37°C for 20 min, and the amount of surface receptors remaining was quantified using ELISA. P2Y_1_ surface expression and ADP induced internalization was comparable in both the control cells and ALIX depleted cells (Fig 6B), suggesting that ALIX does not regulate P2Y_1_ internalization. To examine whether ALIX regulates P2Y_1_ lysosomal degradation, HeLa cells co-transfected with FLAG-P2Y_1_ WT and non-specific or ALIX siRNAs were stimulated with ADP for 180 min, and the extent of P2Y_1_ degradation was analyzed by immunoblotting. Depletion of ALIX blocked ADP-dependent P2Y_1_ WT degradation, with only a 10% loss of receptor (Fig 6C, lane 4), whereas ~50% receptor degradation was observed in control non-specific siRNA transfected cells (Fig 6C, lane 2). These results suggest that ALIX mediates P2Y_1_ lysosomal degradation. We next used a proteinase-K protection assay to examine whether ALIX is required for P2Y_1_ sorting into intraluminal vesicles of multivesicular endosomes. HeLa cells transfected with P2Y_1_-WT and either non-specific or ALIX siRNAs were pre-treated with 2 mM leupeptin and then stimulated with 10 μM ADP for 180 min. Cells were gently permeabilized with digitonin and equivalent amounts of cell membranes were treated with H_2_O (Ctrl), Proteinase-K alone, or Proteinase-K in 1% Triton X-100. In cells treated with non-specific siRNA, ~75% of P2Y_1_ is degraded by proteinase K at steady-state (Fig 6D, lane 2), indicating that the majority of the receptor is basally expressed at the plasma membrane and/or the limiting membranes of endosomes. Upon stimulation with ADP, ~50% of receptors are sorted into protective endosomal compartments (Fig 6D, lane 5). However, in cells depleted of ALIX, ~75% of P2Y_1_ is sensitive to proteinase-K activity at both steady-state and following ligand stimulation (Fig 6D, lanes 8 and 11), indicating that ALIX is required for P2Y_1_ sorting into protective endosomal compartments. Together, these results demonstrate that ALIX interacts with P2Y_1_ via the conserved YPX_3_L motif localized in ICL2 to mediate P2Y_1_ lysosomal sorting and degradation (Fig 7).

## Discussion

The lysosomal sorting and degradation of GPCRs is important for proper signaling and appropriate cellular responses [5]. Here, we examined the mechanisms that regulate the lysosomal sorting and degradation of the purinergic GPCR P2Y_1_. Like many GPCRs, we found that P2Y_1_ is ubiquitinated following agonist stimulation and internalization, and mutation of three C-terminal tail lysine residues abolished P2Y_1_ ubiquitination. Ubiquitin is known to function as signal that targets GPCRs to lysosomes, including the β2-adrenergic receptor [36], CXCR4 [28], and PAR2 [37]. However, our results revealed that like the P2Y_1_ wild-type, ADP stimulation of the P2Y_1_ ubiquitin-deficient mutant promoted receptor degradation, consistent with ubiquitin-independent lysosomal sorting. In addition, both the conserved P2Y_1_ YPX_3_L motif and ALIX are required for sorting the receptor to multivesicular bodies/lysosomes for degradation after ADP stimulation, rather than receptor ubiquitination. Together these finding suggest that like PAR1, the P2Y1 receptor utilizes a novel ALIX-dependent and ubiquitin-independent pathway to sort to lysosomes.

There are now multiple examples of GPCRs that undergo lysosomal sorting independent of ubiquitination, including the δ-opioid receptor (DOR) [38, 39] and PAR1 [40]. Although DOR does not require direct ubiquitination prior to lysosomal sorting, DOR degradation requires components of the ESCRT pathway, including the ubiquitin-binding subunit HRS [38]. In addition, agonist stimulation of DOR promotes interaction with the adaptor protein GPCR-associated sorting protein-1 (GASP-1), which binds to the C-terminal tail [41], although the precise mechanism of interaction is not known [42]. GASP-1 then binds to dysbindin, which interacts with HRS and permits DOR to sort through the canonical ESCRT pathway [43]. In comparison to DOR, the C-terminal tail of PAR1 does not interact robustly with GASP-1 [42]. In addition, PAR1 ubiquitin-independent degradation does not require HRS or entry into the canonical ESCRT pathway [31]. Although the P2Y_1_ C-terminal tail was not included in the original screen for GPCRs that interact with GASP-1 [42], our study demonstrates that HRS is not required for the degradation of P2Y_1_. These results suggest that P2Y_1_ sorts to lysosomes via an alternate pathway that does not require entry into the canonical ESCRT pathway.

The lysosomal sorting pathways of both DOR and PAR1 converge at the ESCRT-III complex and the AAA-ATPase Vps4 [4, 38]. The ESCRT-III complex oligomerizes on the cytoplasmic surface of late endosomes at the intraluminal vesicle bud and facilitates the final closure and scission of the ILV into the lumen of the endosome [18, 44]. Vps4 regulates ILV scission during receptor lysosomal sorting by recycling of ESCRT-III complex components [45, 46]. Expression of a Vps4 E228Q dominant-negative mutant is sufficient to inhibit the degradation of DOR [38] and PAR1 [4], as well as ubiquitinated GPCRs including CXCR4 [28] and PAR-2 [4]. Consistent with these findings, expression of the Vps4 E228Q mutant blocks P2Y_1_ degradation. These results support a model in which multiple pathways exist to facilitate GPCR sorting from early endosomes to late endosomes where all cargo targeted for degradation is packaged by the ESCRT-III complex and Vps4.

In recent work we showed that the adaptor protein ALIX facilitates the degradation of PAR1 through a novel pathway that bypasses the requirement for direct receptor ubiquitination and ubiquitin-binding ESCRT components [4]. Instead, ALIX binds to a YPX_3_L motif within the PAR1 ICL2, which is required for PAR1 lysosomal sorting [4]. P2Y_1_ also contains highly conserved YPX_3_L motifs within ICL2, and we showed that mutation of this motif inhibits P2Y_1_ degradation and interaction with ALIX. In addition, ALIX depletion blocks P2Y_1_ degradation. These findings demonstrate a novel role for ALIX in the regulation of P2Y_1_ and suggest that ALIX regulates ubiquitin- and HRS- independent lysosomal sorting of a subset of mammalian GPCRs.

Unlike PAR1 which sorts directly to lysosomes following stimulation, ADP-stimulated P2Y_1_ initially recycles from endosomes to the plasma membrane and only sorts to lysosomes upon prolonged activation. P2Y_1_ recycling is mediated by sorting nexin-1 (SNX-1) [14], however the mechanisms that regulate receptor sorting into SNX1 endosomal microdomains is poorly understood. One possible mechanism may involve regulation of SNX1 itself. SNX1 binds to endosomal membranes via its Bin/Amphiphysin/Rvs (BAR) domain which induces endosomal membrane curvature [47]. Recent findings have also shown that the endocytic protein RME-8, a modulator of branched actin formation, regulates SNX1 tubule formation [48], however whether GPCR signaling regulates SNX1 membrane association or RME-8 is not known.

Alternatively, prolonged P2Y_1_ signaling may directly regulate ALIX. ALIX facilitates GPCR sorting into intraluminal vesicles of multivesicular endosomes by coupling receptors to the ESCRT-III complex [4]. The N-terminal Bro1 domain of ALIX binds to the ESCRT-III complex subunit CHMP4 [49] and facilitates ALIX interaction with endosomal membranes [50]. ALIX interacts with YPX_3_L motifs in target receptors and other cargo via a central V domain [4, 51]. In addition, recent studies demonstrate that ALIX is modified with ubiquitination during the lysosomal sorting of PAR1 [52]. The alpha arrestin ARRDC3 recruits the E3 ubiquitin ligase WWP2 to ubiquitinate ALIX following stimulation of PAR1. Inhibition of ARRDC3 or WWP2 expression using RNA interference is sufficient to block ALIX ubiquitination as well as PAR1 degradation [52], suggesting that ubiquitination of ALIX is critical for regulating PAR1 sorting at late endosomes. Interestingly, the ALIX V-domain also harbors a novel ubiquitin-binding domain [53, 54], and recent findings suggest that ALIX dimerization is critical for mediating ALIX function [50, 55]. However, the role of the ALIX ubiquitin-binding or dimerization in regulating P2Y_1_ lysosomal sorting is not known. Future studies will focus on elucidating the mechanisms that regulate ALIX in response to GPCR activation, including whether P2Y_1_ and PAR1 signal to ALIX through convergent pathways.

Our findings suggest that P2Y_1_ does not require receptor ubiquitination for internalization or lysosomal sorting, however, P2Y_1_ becomes ubiquitinated following agonist stimulation and internalization [23]. This raises interesting questions regarding the function of P2Y_1_ ubiquitination. Recent evidence demonstrates that ubiquitination of P2Y_1_ is required to promote activation of the p38 mitogen-activated protein kinase (MAPK) [23]. Ubiquitin-mediated induction of p38 MAPK by P2Y_1_ occurs through a non-canonical pathway mediated by transforming growth factor beta-activated kinase-binding protein-1 (TAB1). Depletion of TAB1 or mutation of P2Y_1_ ubiquitination sites inhibits p38 MAPK activation. Interestingly, the TAB1-p38 MAPK signaling complex appears to assemble on endosomes, supporting a model in which GPCRs may activate signaling effectors from endosomes [56]. In addition, many of the GPCRs that contain YPX_3_L motifs have been implicated in inflammatory signaling [4]. Thus, further investigation is required to determine whether these GPCRs also signal via a ubiquitin-dependent mechanism to p38 MAP kinase or other signaling pathways. However, the possibility exists that a wide range of GPCRs signal via TAB1 and TAB2, and are regulated by ALIX.

In summary, this study demonstrates a novel role for the adaptor protein ALIX in the ubiquitin-independent degradation of the activated P2Y_1_ purinergic receptor. We have shown that P2Y_1_ lysosomal sorting does not require receptor ubiquitination or the ubiquitin-binding ESCRT subunit HRS. Instead, the degradation of P2Y_1_ requires a conserved YPX_3_L motif located within ICL2. The P2Y_1_ YPX_3_L motif is required for receptor sorting into the lumen of multivesicular bodies and for binding to the adaptor protein ALIX. ALIX expression is also required for agonist-induced P2Y_1_ degradation. Our data support the hypothesis that ALIX mediates the lysosomal sorting of a subset of GPCRs that harbor YPX_3_L motifs within the intracellular loops of the receptor.
